# Knowledge, Perception, and Attitude Toward Voluntary Counseling and Testing for HIV Among Secondary School Students in Iringa Rural District: Descriptive Cross-Sectional Study

**DOI:** 10.2196/66739

**Published:** 2025-09-26

**Authors:** Maiko Charles Mkwambe, Shakilu Jumanne Kayungo, Youping Deng

**Affiliations:** 1 Department of Pediatrics, Zhongnan Hospital of Wuhan University, 169 Donghu Road, Wuchang District, Wuhan, Hubei, 430071, China, 86 27-67811887; 2 Department of Paediatrics & Child Health, University of Dodoma, Dodoma, United Republic of Tanzania

**Keywords:** knowledge, perception, attitudes, secondary school students, voluntary counselling and testing, HIV, AIDS, sexually transmitted diseases, questionnaire, quantitative, cross-sectional

## Abstract

**Background:**

Voluntary counseling and testing (VCT) for HIV/AIDS is characterized by several key components, including pretest and posttest counseling, as well as the formulation of individualized risk reduction plans. Adolescents, including secondary school students, represent a population particularly vulnerable to HIV infection due to various biological, psychological, and social factors.

**Objective:**

The present study aimed to assess the knowledge, perception, attitude, and barriers toward VCT for HIV/AIDS among secondary school students in Iringa Rural District, Tanzania.

**Methods:**

A descriptive cross-sectional study was conducted in Iringa Rural District, targeting secondary school students. A random sampling technique was employed to select the participating schools. Data were collected through self-administered questionnaires, which were completed solely by students who voluntarily consented to participate in the study. Data management and analysis were carried out using Epi Info™ 7.2 software.

**Results:**

The study involved 127 secondary school students aged 15‐25 years from three schools in Iringa Rural District, with 69 (54.3%) female and 58 (45.7%) male participants. All students were aware of the VCT services. The primary source of VCT information was school-based education (92, 33.3%), followed by radio/TV (65, 23.6%), friends/family (46, 16.7%), and magazines (35, 12.7%). Some students also cited health centers, hospitals, and religious seminars (38, 13.8%) as sources. Knowledge of VCT increased with education level, with Form IV students showing the highest awareness (67, 55.4%). Most students understood VCT’s main purpose: 88 (50.6%) of them linked it to knowing one’s HIV status, 58 (33.3%) to HIV prevention, and 28 (16.1%) to preparing for test results. No misconceptions about VCT’s purpose were reported. Attitudes toward HIV testing showed that stigma concerns increased with age, particularly among those aged 17‐18 years, where 26 (60.5%) were unwilling to be identified as HIV-positive. Nonetheless, 65 (51.2%) students supported knowing their HIV status and 86 (54.4%) said they would inform others and change their behavior after testing. Key barriers to VCT uptake included fear of stigma (71, 50.6%), lack of confidentiality, insufficient trained personnel, and poor infrastructure.

**Conclusions:**

The study found that secondary school students in Iringa Rural District had generally high awareness and positive attitudes toward VCT, mainly informed through schools and media. Awareness increased with education level, but willingness to disclose test results remained low due to stigma and confidentiality concerns. While many students were ready to take responsible actions after testing, barriers such as fear, misinformation, and limited access to services persisted. The study emphasizes the need for improved school-based VCT programs, better-trained staff, community education, and strategies to address stigma and infrastructural gaps.

## Introduction

Since the year 2000, approximately 38.1 million individuals have contracted HIV, and about 25.3 million have lost their lives due to illnesses related to AIDS. Despite better access to antiretroviral therapy and health care in many parts of the world, the AIDS epidemic still claimed 1.2 million lives in 2014 alone, and globally, youth aged 15‐24 years account for nearly one-third of new HIV infections [1,2]. Despite this, uptake of voluntary counseling and testing (VCT) services among youth remains low. The majority of these cases occurred in sub-Saharan Africa, which is home to 66.6% of all people living with HIV, and most HIV transmissions happen through heterosexual sex, from mother to child during childbirth or breastfeeding, and through unsafe blood transfusions [1,3]. Moreover, since there is no effective vaccine or cure, VCT for HIV has served as a key entry point, providing access to a range of prevention and care services, including antiretroviral therapy.

Sub-Saharan Africa’s burden is particularly evident in countries like Tanzania, where rural regions face distinct challenges. HIV/AIDS remains a major public health concern in Tanzania, with approximately 1.6 million people living with HIV as of 2023 [4]. While the national prevalence stands at 4.7% among adults aged 15‐49 years [5], some regions, particularly in the southern highlands, report significantly higher rates. Iringa Region, including its rural districts, has one of the highest HIV prevalence rates in the country, reported at 11.1% after Njombe region, which is the leading region with highest HIV infection of 12.3% [6]. This high prevalence underscores the need for targeted interventions, especially among vulnerable populations such as secondary school students. Rural communities in Tanzania experience unique challenges related to HIV, including limited access to health care services, stigma, and lower awareness levels [7]. These issues are compounded by traditional gender norms, low literacy rates, and poverty, which hinder HIV prevention and testing efforts [8].

VCT is a critical component in HIV prevention and control strategies. However, its uptake among secondary school students in Tanzania remains suboptimal. Studies have shown that while awareness of VCT services is relatively high, actual utilization is low due to factors such as fear of positive results, stigma, and lack of perceived risk [9,10]. In Iringa Rural District, specific data on VCT uptake among secondary school students is limited, highlighting the need for focused research in this demographic to inform targeted interventions. Adolescents and young people, particularly those in secondary schools, represent a vulnerable group in the HIV epidemic. In rural Tanzania, multiple structural, social, and cultural factors inhibit youth from accessing VCT.

A predominant barrier to VCT uptake is the fear of receiving a positive HIV result, which is often associated with social stigma. Students express concerns about being ostracized by peers and community members if they tested positive, leading to reluctance in seeking testing services. This fear is compounded by misconceptions about HIV transmission and the belief that a positive result equates to imminent death [11-13]. Students often worry about the confidentiality of their test results, fearing that health workers may disclose their status to others in the community. This concern is particularly acute in tight-knit rural communities where privacy is hard to maintain. Ensuring strict confidentiality protocols and training for health workers is crucial. In tight-knit rural communities, concerns about privacy can discourage individuals from utilizing local testing services [11].

The absence of youth-friendly VCT services tailored to adolescents' needs and concerns contributes to low uptake. Health facilities may not provide an environment that is welcoming or comfortable for young people, deterring them from seeking services. Implementing adolescent-focused approaches and involving youth in service design can enhance engagement. Many health facilities are not tailored to meet the specific needs of adolescents, making them less likely to seek testing [11]. Geographical barriers and inadequate health care infrastructure in rural areas hinder access to testing facilities [14]. In rural districts like Iringa, access to VCT services is hindered by geographical barriers, including long distances to health facilities and lack of transportation. Additionally, the operating hours of VCT centers may not align with students’ schedules, further limiting accessibility. A significant number of youths underestimate their risk of HIV infection, leading to lower testing rates. Many secondary school students in rural Tanzania perceive themselves to be at low risk of HIV infection, even when engaging in high-risk behaviors. This low-risk perception diminishes the perceived need for testing, thereby reducing VCT uptake [11,15]. Similar youth-focused barriers have been reported in other low-resource settings, underscoring the global challenge of VCT uptake [16-18].

Employing diverse strategies to fight against HIV and minimize its effects on individuals, families, and society is crucial. These approaches encompass VCT, provider-initiated counseling and testing, diagnosing HIV in infants and young children, providing family care and partner testing, detecting and managing sexually transmitted infections, offering counseling on safer sex and risk reduction, promoting male circumcision, and implementing targeted interventions for sex workers and individuals who identify as homosexual [19].

While there is some data on the awareness and usage of VCT services in a few regions in Tanzania, there is a noticeable gap in research regarding the knowledge, attitudes, and perceptions of secondary school students toward VCT services. This is worrisome because the secondary school setting is conducive to engaging in risky HIV behaviors, like unprotected sex. Secondary school students are especially susceptible to these behaviors due to their inclination toward sexual experimentation, involvement with multiple partners, and inconsistent condom use [20].

To our knowledge, there is a lack of studies assessing knowledge, attitudes, and perceptions among secondary school students in Iringa Rural District. Also, the provision of VCT services in Iringa has been inconsistent, and even when these services are accessible, the number of people utilizing these, especially youths, has been relatively low. To ensure effective VCT services, it was essential to evaluate the awareness, usage, and willingness of secondary school students toward VCT services. Consequently, this study focused on examining the knowledge, attitudes, and perceptions of secondary school students toward VCT services in the Iringa Rural District in the Iringa region.

## Methods

### Study Setting and Study Design

This study employed a cross-sectional descriptive study to assess the knowledge, perception, and attitude toward VCT among secondary school students in Iringa Rural District. This study was done at three secondary schools (Lipuli, Kalenga, and Kidamali) from September to December 2023. Iringa Rural District is one of the four districts of the Iringa Region of Tanzania, East Africa. It is bordered to the north by the Dodoma Region, to the east by Kilolo District and encircles Iringa Urban District, to the south by the Mufindi District, to the southwest by the Mbeya Region, and to the northwest by the Singida Region. Iringa district has a cold climate, which favors agricultural activities and livestock keeping; thus, the majority of the people are peasant farmers. Original occupants are the Hehe tribe, but due to the migration of people, the district has been occupied by people of different ethnicity.

### Study Population

The target population was students from three secondary schools and comprised both males and females aged between 15 and 25 years. This age group was chosen due to their vulnerability to HIV infection and the relevance of VCT in their health-seeking behavior.

### Sample Size

Sample size was calculated from the formula N=Z^2^PQ/E^2^, where N=minimum sample size; Z=standard normal deviation, usually set at 1.96, which corresponds to 95% CI; Q=(1–P); P=the prevalence of HIV/AIDS in Iringa region (9.1%) [Tanzania HIV Impact Survey (THIS 2022)–National AIDS Control Programme, Tanzania [21], and US President’S Emergency Plan for AIDS Relief (PEPFAR) Tanzania Country Operational Plan (COP 2023)–PEPFAR [22]], E=standard error of estimate, 0.05. Therefore, the sample size required was 127.

### Sampling Technique

A multistage sampling technique was used. Stage 1: Three wards were randomly selected from Iringa Rural District. Stage 2: From each ward, one public secondary school was selected using simple random sampling. Stage 3: Within each school, a list of eligible students was obtained and systematic random sampling was used to select participants proportional to the school size.

### Inclusion Criteria

The inclusion criteria for the study were secondary school students, aged between 15 and 25 years, willing to be interviewed and participate in the study, enrolled in Form I-IV.

### Exclusion Criteria

This study excluded students who were below 15 years and above 25 years of age, who declined to participate in the study, who were absent on the day of data collection, and who had cognitive impairments that limited their ability to participate.

### Data Collection

#### Data Collection Tools

The data were collected from secondary school students using self-administered questionnaires. The questionnaire was developed based on validated tools from similar studies and included the following sections: demographics (age, sex, grade level), knowledge on HIV and VCT, perception of risk and benefits of VCT, attitudes toward VCT services, and barriers to accessing VCT services. The questionnaire was pretested among 20 students from a school not included in the main sample to assess clarity and reliability.

#### Data Collection Procedure

Data collection was conducted by trained research assistants fluent in both Kiswahili and English. Prior to administration, participants were informed about the study objectives and provided with instructions, allowing them to make an informed decision about their own participation. Their questions were addressed. Participants were informed that their involvement in the study was voluntary. To ensure anonymity, participants were instructed not to provide their names on the questionnaire forms. Confidentiality was also assured and confidentiality guarantees given.

### Data Entry and Analysis

Coding, entry, cleaning, and analysis of data were done using Epi Info™ 7.2 software (Centers for Disease Control and Prevention). Descriptive statistics (frequencies and percentages) were computed.

### Ethical Considerations

Ethical approval of the study was obtained from the Review Board of the University of Dodoma (DJ.232/238/0‐28) and Iringa district council (FA.255/265/01/PART ‘C;/72), with a waiver of written informed consent. To maintain confidentiality, unique identification codes were assigned to the study subjects. The principal investigators securely stored and controlled access to the data, limiting it to authorized personnel only. The study was performed in accordance with the University of Dodoma ethical guidelines for research involving human subjects and the ethical standards of the 1964 Declaration of Helsinki and its later amendments or comparable ethical standards.

## Results

### Characteristics of the Study Participants

A total of 127 students from three secondary schools aged 15‐25 years were enrolled in the study. Out of the 127 students, 69 (54.3%) and 58 (45.7%) students were females and males, respectively. Participants were mostly within the adolescent age group. A majority of 72 (56.7%) students were aged 17‐18 years, followed by 41 (32.3%) aged 15‐16 years, and only 14 (11%) aged 19‐20 years. This age range is particularly significant, as adolescents are a key population for HIV prevention strategies due to their increased vulnerability and evolving risk behaviors. In terms of academic level, the largest proportion of students 70 (55.1%) were in Form IV, followed by Form III 48 (37.8%), and a small group 9 (7.1%) in Form II. This distribution indicates that most participants were senior students who are likely to have greater exposure to sexual and reproductive health education. All the students were aware of VCT services. The source of information on VCT for HIV/AIDS among students was determined. The primary source of information that involved describing whether they have heard of VCT or not was their schools, accounting for 92 (33.3%) participants. Radios or TV were the second common source, with 65 (33.3%) participants obtaining information from this medium. Friends or family were another significant source, with 46 (16.7%) participants relying on them. Magazines were the least utilized source, with only 35 (12.7%) participants obtaining information from them. On the other hand, approximately 38 (13.8%) participants reported obtaining information from health centers, hospitals, mosques, and church seminars (Table 1).

**Table 1.** Demographic characteristics of the study group.

Characteristics	n (%)
Gender	
Female	69 (54.3)
Male	58 (45.7)
Age (years)	
15-16	41 (32.3)
17-18	72 (56.7)
19-20	14 (11.0)
Grade	
II	9 (7.1)
III	48 (37.8)
IV	70 (55.1)
Student knowledge toward VCT^a^ services	
Yes	127 (100)
No	-
Source of information about VCT services	
Friends/family	46 (16.7)
Taught in school	92 (33.3)
Radios/TV	65 (33.3)
Magazine	35 (12.7)
Others	38 (13.8)

a VCT: voluntary counseling and testing.

### Knowledge Toward the Importance of VCT Services

Table 2 and Table 3 present two key elements: (1) the level of students’ awareness of the importance of VCT services by education level and (2) students’ understanding of specific reasons why VCT services are important.

**Table 2.** The students’ knowledge about the importance of VCT^a^ services (N=127). The categorical variables are represented in numbers and percentages.

Level of education	Do you know the importance of VCT services? n (%)	
	Yes	No
Form II	8 (6.6)	1 (16.7)
Form III	46 (38.0)	2 (33.3)
Form IV	67 (55.4)	3 (50.0)

a VCT: voluntary counseling and testing.

**Table 3.** Perceived importance of VCT^a^ services (N=127). The categorical variables are represented in numbers and percentages.

Importance of VCT services	n (%)
To know HIV status	88 (50.6)
Prepares you to accept HIV test results	28 (16.1)
Important in HIV prevention	58 (33.3)
Stigmatization to infected	-
Confuse infected person	-

a VCT: voluntary counseling and testing.

### Awareness of the Importance of VCT by Education Level

The distribution of responses by form level indicates a trend of increasing awareness with educational progression. Among Form II students, 8 out of 9 (88.9%) reported knowing the importance of VCT services, while 1 (11.1%) student did not. In Form III, 46 (95.8%) students demonstrated awareness, with only 2 (4.2%) students responding “No.” Among Form IV students, the highest level of awareness was observed, with 67 (95.7%) students, and only 3 (4.3%) students reporting no awareness (Table 2 and Table 3).

### Perceived Importance of VCT Services

When asked about why VCT is important, students identified three major reasons: (1) To know HIV status (88, 50.6%); this was the most frequently cited reason. (2) Important in HIV prevention (58, 33.3%), significant proportion recognized VCT’s preventive value. (3) Prepares you to accept HIV test results (28, 16.1%); this shows that some students understood the emotional or psychological support role that VCT provides, though this was less frequently mentioned. No students associated VCT with negative outcomes such as stigmatization or confusing the infected person (Table 2 and Table 3).

### Attitudes of Participants Towards VCT Services of Secondary School Students

Regarding attitudes of secondary school students toward uptake of VCT, Table 4 shows three dimensions: (1) readiness to be known for their HIV status by age, (2) general acceptability of HIV testing, and (3) willingness to disclose HIV test results.

**Table 4.** Attitudes of secondary school students toward HIV tests (N=127). The categorical variables were represented in numbers and percentages.

	Acceptance level, n (%)				
Attitudes	Strongly disagree	Disagree	Agree	Strongly agree	Not sure
Are you ready to be known for your HIV status?					
Age (years)					
15‐16	12 (27.9)	11 (40.7)	5 (22.7)	9 (33.3)	4 (50.0)
17‐18	26 (60.5)	12 (44.4)	15 (68.2)	15 (55.6)	4 (50.0)
19‐20	5 (11.6)	4 (14.8)	2 (9.1)	3 (11.1)	—^a^
Acceptability of secondary school students towards HIV test	6 (4.7)	8 (6.3)	40 (31.5)	65 **(**51.2)	8 (6.3)
Willingness of secondary school students to disclose the HIV test results	43 (33.9)	27 (21.3)	22 (17.3)	27 (21.3)	8 (6.3)

a not applicable.

### Readiness to Be Known for Their HIV Status By Age

Responses varied across age groups, with the 17‐ to 18-year-old group showing the highest level of acceptance; among those who agreed or strongly agreed to being known for their HIV status, the 17‐ to 18-year-old group made up the majority, accounting for 15 (68.2%) agreeing and 15 (55.6%) strongly agreeing. In contrast, the 15‐ to 16-year-old group had relatively higher percentages in disagreement or uncertainty, where 12 (27.9%) strongly disagreed, 11 (40.7%) disagreed, and 4 (50%) were not sure. The 19‐ to 20-year age group showed lower engagement across all categories (Table 4).

### Acceptability of HIV Testing

Overall, the acceptability of HIV testing among students was encouraging, where a large majority either agreed (40, 31.5%) or strongly agreed (65, 51.2%) with the idea of HIV testing. Only 14 (11%) (6 strongly disagree +8 disagree) rejected the idea of testing, and 8 (6.3%) were unsure. This positive attitude toward testing reflects a generally favorable view of VCT among the student population, showing potential for increased VCT uptake (Table 4).

### Willingness to Disclose HIV Test Results

When it comes to disclosing HIV status, students were less willing; out of 127 students, a significant proportion of 43 (33.9%) strongly disagreed and 27 (21.3%) disagreed with the idea of disclosing their HIV test results. However, only 22 (17.3%) agreed and 27 (21.3%) strongly agreed to disclose their results, while 8 (6.3%) remained unsure, indicating a substantial level of concern over confidentiality, stigma, or social repercussions associated with a known HIV status—even among those who accept testing (Table 4).

### Perception of Participants Toward VCT Services of Secondary School Students

Insights into students’ anticipated actions after receiving HIV test results and their expected reactions if they tested HIV-positive were determined. These perceptions reflected both their understanding of the implications of testing and their emotional and behavioral readiness to cope with outcomes.

### Actions to Be Taken After Testing for HIV

Out of 150 responses, 86 (54.4%) students responded that they will tell their parents or close friends, indicating a relatively positive perception of social support. Also, 50 (31.7%) students responded to changing behavior. This significant proportion of students reported that they would modify their behavior after an HIV test, which is a highly encouraging sign. While 14 (8.9%) students reported using condoms, reflecting that awareness or acceptance of specific protective practices like condom use may still be limited. Cultural taboos or misinformation may be contributing factors. However, 8 (5.1%) students responded not telling anyone (Table 5). A minority of students indicated they would remain silent about their results. This may reflect concerns about stigma, fear of discrimination, or lack of trust in confidentiality.

**Table 5.** Perception of secondary school students toward VCT^a^ services for HIV testing (N=127). The categorical variables were represented in numbers and percentages.

Variable	n (%)
Action to be taken by secondary school students after having tested for HIV	
I will tell my parents/close friends	86 (54.4)
I won’t tell anybody	8 (5.1)
I will change my behavior	50 (31.7)
I will start using condom	14 (8.9)
Students’ reaction if tested positive for HIV	
Kill myself	1 (0.5)
Return to God	14 (7.4)
Follow doctor’s order	122 (64.2)
Inform my parents/close friends	23 (12.1)
Continue with my normal life	14 (7.4)
Others	—^b^

a VCT: voluntary counseling and testing.

b not applicable.

### Reactions if Tested Positive for HIV

Out of 174 students’ responses, 122 (64.2%) students responded to follow doctor’s orders, the overwhelmingly dominant response showing a strong level of health-seeking behavior and trust in medical professionals. Also, 23 (12.1%) students showed that they will inform their parents or close friends, showing a willingness by some to seek emotional support and maintain open communication. Returning to God was reported by 14 (7.4%) students, and 14 (7.4%) students reported continuing with normal life, indicating spiritual and psychological coping mechanisms.

Suicidal ideation was reported by 1 (0.5%) student; the fact that even one student reported this highlights the psychological distress and fear that can be associated with an HIV-positive diagnosis. It underscores the importance of counseling services and mental health support within VCT programs (Table 5).

### Barriers Toward Not Using VCT Services Among Secondary School Students

Barriers among secondary school students toward utilization of VCT services were determined. Out of 140 students’ responses, most students (71, 50.6%) responded that stigmatization from society was the great obstacle to having an HIV test. While 26 (18.6%) students said that they will die early, reflecting fear-based misconceptions about HIV. On the other hand, 25 (17.86%) students said that a lack of confidentiality still exists among health care workers, making it harder for them to know their HIV statuses. Thirteen (9.3%) students believed that they are too young to get HIV, reflecting low perceived susceptibility. However, 5 (3.6%) students reported perceived cost of VCT services, reflecting that VCT services are usually free in Tanzania (Table 6).

**Table 6.** Barriers among secondary school students towards utilization of VCT^a^ services (N=127). The categorical variables were represented in numbers and percentages.

Variable	n (%)
Barriers to students not to use VCT services	
Stigmatization	71 (50.6)
I’m very young; therefore, I can’t get HIV	13 (9.3)
I will die early	26 (18.6)
VCT services are expensive	5 (3.6)
Lack of confidentiality among health workers	25 (17.9)
Reason for lack of coverage of VCT services for secondary school students	
Poor infrastructure	17 (21.1)
Lack of personnel for VCT services	46 (56.8)
I don’t want this service	2 (2.5)
It brings conflicts among people	9 (11.1)
I don’t believe in this service	2 (2.5)
Others	5 (6.2)

a VCT: voluntary counseling and testing.

### Reasons for Lack of VCT Service Coverage for Students

Reasons for lack of coverage for VCT services for secondary school students were determined. Out of 81 total responses of students, 46 (56.8%) students reported lack of personnel response, revealing a critical shortage of trained staff to provide VCT in schools or nearby health centers. Poor infrastructure was reported by 17 (21.1%) students, reflecting a broader health systems challenge. Conflicts among people were reported by 9 (11.1%) students, and mistrust and disinterest were reported by 2 (2.5% each) students, suggesting low health literacy or resistance to external influence (Table 6).

## Discussion

### The Main Findings

Accurately measuring VCT service uptake among young adults is imperative and essential, not only due to their heightened vulnerability to HIV infection but also because this population frequently encounters significant structural and socioeconomic impediments to accessing these critical services. The findings reveal a critical paradox; although student awareness of VCT services was widespread and views on its importance were predominantly positive, alongside strong endorsement for the acceptability of HIV testing itself, significant barriers persist that account for the low utilization rates observed in Iringa Rural District. Among these barriers are a pronounced reluctance to disclose HIV status, rooted primarily in fear of societal stigmatization, concerns about confidentiality breaches, and prevalent misconceptions regarding HIV/AIDS and VCT outcomes. Furthermore, systemic challenges, notably severe shortages of trained personnel and inadequate infrastructure, directly contribute to the inconsistent VCT service provision in Iringa Rural District. These findings collectively demonstrate that high awareness is necessary but insufficient for effective VCT uptake.

### Participant Characteristics and Implications for VCT Uptake

This study enrolled 127 secondary school students aged 15‐25 years from three secondary schools, with a relatively balanced gender distribution, consistent with school-based demographic trends in rural Tanzania. The majority were aged 17‐18 years, suggesting that mid-adolescence is the key period for interventions promoting VCT services. This age group typically experiences increased autonomy, sexual debut, and curiosity—factors that elevate HIV risk and underline the importance of early preventive strategies. Similar demographic trends were found in a study conducted by Mkumbo and Ingham in Mwanza [23], which emphasized that students aged 16‐18 years were more receptive to sexual and reproductive health interventions due to greater maturity and exposure to sexual reproductive health topics in school curricula. This supports the current finding that students in higher academic levels (Form III and IV) showed greater awareness and more positive attitudes toward VCT, emphasizing the educational system’s critical role in shaping health behaviors.

### Knowledge of VCT and Information Sources

All students reported awareness of VCT services, and schools were identified as the main source of this information, followed by radio/TV and friends/family. This aligns with findings by Anastasia et al in Uganda, where school-based interventions were the leading source of HIV-related knowledge among adolescents [24]. The prominence of mass media as a secondary source supports the notion that integrating media campaigns with school-based programs can reinforce HIV prevention messages. However, the low reliance on interpersonal sources like family or health centers suggests a missed opportunity for community-based health promotion. This is consistent with Mahmoud et al who reported that youth often perceive HIV as a “school topic” rather than a family concern, emphasizing the need to involve parents and local health care workers in sensitization campaigns to bridge this communication gap [25]. Our study findings were different from the study conducted by Shedura et al, who found that most of the students did not know about VCT services for HIV/AIDS. Moreover, 157 (68.0%) students have never heard of VCT, while only 74 (32.0%) students have heard of VCT, but higher findings from mass media by the study conducted by Sisay et al found that the most frequently mentioned sources of information for HIV/AIDS were mass media 211 (62.2%) followed by health professionals 177 (52.2%) [26,27]. This signifies that our study found a very low rate of VCT services information from mass media by secondary school students. Our study showed that there is a need to develop a policy that will be beneficial to have VCT training programs and seminars in all mass media in Tanzania on VCT education, so as to facilitate the awareness of VCT services for HIV/AIDS among youths.

Awareness of the importance of VCT improved with educational level, with Form IV students demonstrating the highest levels of awareness. This echoes findings from Mboya et al in Southern Highlands, Tanzania, where older students and those in upper forms exhibited significantly higher knowledge and appreciation of VCT’s role in HIV prevention and care [28]. Students primarily cited knowing one’s HIV status and preventing HIV as key reasons for seeking VCT, indicating a sound understanding of VCT’s core objectives. Nevertheless, the relatively low mention of emotional preparedness reflects a gap in psychological aspects of VCT counseling. Comprehensive VCT services should therefore include components that prepare youth emotionally, not just medically, for their HIV test results. Similarly, a study by Mshana et al in rural Tanzania found that upper-secondary students (Forms III–IV) had significantly higher VCT knowledge than Form I students, attributing this to cumulative exposure to HIV education in school curricula [10]. In Kenya, Maitino et al observed that Form IV students were twice as likely to accurately describe VCT benefits compared to Form I peers, linking this to peer-led interventions in higher grades [29]. A Ugandan study by Nabaggala et al reported similar trends, with senior students (aged 17‐19 years) showing 95% awareness of VCT versus 68% among juniors, due to repeated sensitization in advanced biology and life-skills classes [30]. Banda et al. in their study showed that, in terms of peer influence, senior students act as information conduits [31]. The consistency of these findings suggests that scaling up age-appropriate, curriculum-based VCT education—particularly in lower secondary levels—could bridge awareness gaps. Pairing this with peer-led initiatives (eg, Form IV ambassadors) may amplify impact.

In terms of anticipated posttest behavior, the most common actions were informing parents or close friends and changing risky behavior. These findings indicate a positive perception of VCT as a preventive and supportive service. However, the low number of students indicating condom use as a posttest action suggests persistent gaps in knowledge or cultural taboos around condom use. A study by Culyba et al in rural Morogoro found that misconceptions and sociocultural norms significantly deterred condom acceptance, even among informed youth [32]. The vast majority of students said they would follow doctors’ orders if tested HIV-positive, which is encouraging. However, the fact that one student reported suicidal ideation highlights the urgent need for school-based mental health services and counseling support integrated into VCT programs. Furthermore, in our study, many respondents cited fear and anxiety as the main reasons for their reluctance to disclose their HIV test results. They worried about the emotional distress and disruption to their lives that could result from a positive test result. This reflects the reason why there is still a prevalence of HIV infection in Iringa, accounting for a second leading region after Njombe region, as most young population who are sexually active continue their inclination toward sexual experimentation, involvement with multiple partners, and inconsistent condom use while fearing to disclose their HIV statuses to their counter sexual partners. The lack of knowledge and confidence in dealing with the outcome, as well as uncertainty about posttest support and care, contributed to their concerns. These findings are consistent with a study conducted in Ethiopia, which also identified fear and anxiety as influential factors in the uptake of VCT among young people [33,34].

The majority of students expressed positive attitudes toward HIV testing, with 51.2% strongly agreeing and 31.5% agreeing to be tested. However, only 38.6% were willing to disclose their HIV test results, while 55.2% disagreed or strongly disagreed. Furthermore, the fact that even one student reported suicidal ideation highlights the psychological distress and fear that can be associated with an HIV-positive diagnosis. It reveals an important barrier to open dialog and follow-up care and highlights the need for strengthening counseling services, promoting confidential testing environments, and conducting anti-stigma campaigns within schools and communities and highlighting the need for follow-up care. It underscores the importance of counseling services and mental health support within VCT programs. This dichotomy reflects a tension between individual health behavior and social repercussions, likely fueled by ongoing stigma. Comparable concerns were found in Wandera et al in Kenya, where stigma was a major deterrent to disclosure despite high testing willingness [35]. Similarly, students who perceived high community stigma were 5 times more likely to refuse disclosure, even if they tested [31]. In Zimbabwe, Fulton et al, in their study, reported that school-based stigma reduction programs increased willingness to disclose by 22%, highlighting the need for psychosocial support in VCT services [36].

The 17‐ to 18-year-old group exhibited the highest acceptance of being known for their HIV status, suggesting this group may serve as peer educators or ambassadors in youth-centered VCT campaigns. On the contrary, the 15‐ to 16-year-old group showed higher levels of uncertainty and fear—highlighting the importance of age-specific sensitization. Older students (19‐25 y) may exhibit greater reluctance due to heightened awareness of social stigma or fear of impacts on future opportunities, necessitating targeted interventions such as private counseling sessions. The findings from this study align with a study in South Africa by Owen et al, who found that 17‐ to 19-year-olds were more likely to test for HIV and disclose their status than younger adolescents, attributing this to increased maturity and sexual health awareness [37]. Research in Kenya by Maitino et al highlighted that peer educators aged 16‐18 years significantly improved VCT uptake in schools, supporting the idea that this age group can effectively influence testing behaviors [29]. Similarly, studies have reported that 15‐ to 16-year-olds often exhibit higher HIV testing hesitancy due to fear of parental disclosure or lack of autonomy. Younger adolescents required school-based sensitization programs to reduce misconceptions, mirroring this study’s call for age-tailored interventions [10,38]. Older youth (19‐25 y) frequently avoid testing due to anticipated stigma, particularly in settings where HIV status can affect employment or relationships [39]. Confidentiality-guaranteed services, such as mobile or after-hours clinics, have proven effective for this group [40].

### Barriers Toward Utilization of VCT Services by Secondary School Students

In this study, Stigma emerged as the most reported barrier, consistent with nearly all recent adolescent HIV studies in sub-Saharan Africa. For instance, Mugisha et al in Uganda reported that perceived stigma was the leading reason adolescents avoided VCT services, despite widespread awareness [41]. In the current study, students also cited fear of dying and concerns about confidentiality, indicating that both misinformation and systemic trust issues with health care workers remain significant challenges, and the belief that they are expensive may indicate poor communication about service accessibility. These findings support the need for community-wide destigmatization campaigns and robust training for health care providers in confidentiality. Similar findings were found by Shedura et al [26], Anaba et al [9], and Adulo et al [42]. Lack of confidentiality among health care providers was also the hindering factor for secondary school students toward VCT services utilization, while others have the notion that they are too young; therefore, they cannot get HIV, which in fact contributes to the continuous prevailing of high spread of HIV infection among young population in the Iringa region.

Our study also identified a lack of trained personnel and poor infrastructure as major constraints to VCT accessibility. These are structural barriers that mirror findings from the Joint United Nations for Acquired Immune Deficiency Syndrome (UNAIDS) (2023), which stressed that youth-friendly HIV services are severely under-resourced in rural areas. Efforts to decentralize and strengthen VCT services in school environments are therefore paramount [43].

### Implications of the Study Findings

#### School-Centered Interventions Are Effective

The high levels of awareness and acceptance of VCT among Form III and IV students affirm the value of integrating VCT education into school curricula. Education ministries should consider expanding such programs to lower forms to reach younger adolescents early.

#### Addressing Stigma and Confidentiality

The reluctance to disclose HIV status and the perceived breach of confidentiality point to urgent needs for stigma-reduction campaigns and the implementation of trusted, youth-friendly VCT centers with strict confidentiality policies.

#### Targeted Behavioral Change Communication

The limited mention of condoms as a preventive method and misconceptions about HIV’s fatality indicate a need for targeted behavioral interventions that challenge myths and promote accurate knowledge, especially around prevention and healthy living.

#### Engaging Families and Communities

Since interpersonal sources like families and health workers play a minor role in VCT education, strategies should involve parents, religious leaders, and community health workers to normalize discussions about HIV and reduce shame.

#### Mental Health Integration

The instance of suicidal ideation underscores the need to include psychosocial support services within VCT frameworks. Adolescents require reassurance and coping mechanisms to handle positive diagnoses constructively.

#### Resource Allocation and Infrastructure

The findings highlight that for VCT programs to scale up in rural districts, investments are needed in human resources, mobile clinics, and infrastructure to bridge current service delivery gaps.

### Limitations of the Study

Although this study provides valuable insights, it recognizes several limitations. This study was conducted in only three secondary schools within Iringa Rural District. The findings may not be generalizable. Future longitudinal research is needed to cover these areas in large geographical populations of multiple regions along the country, compounded with large sample size.

### Conclusion

This study explored the knowledge, perceptions, and attitudes of secondary school students in Iringa Rural District regarding VCT for HIV. The findings revealed a generally high level of awareness and positive attitudes toward VCT services among students, with schools and mass media being the primary sources of information. Awareness of the importance of VCT increased with educational level, reflecting the significant role of school-based health education. Most students recognized the value of VCT in knowing one’s HIV status and preventing the spread of HIV, although fewer acknowledged its psychological support functions. The overall acceptability of VCT was encouraging; however, willingness to disclose test results remained low, underscoring ongoing concerns around confidentiality and stigma. Students demonstrated a readiness to act responsibly upon receiving HIV test results, including informing trusted individuals and adopting safer behaviors. However, some also expressed reluctance to disclose results or misconceptions about HIV, pointing to lingering stigma and misinformation. Key barriers to VCT utilization included societal stigma, fear of early death, perceived lack of confidentiality, and insufficient knowledge. Structural challenges such as a shortage of trained personnel and inadequate infrastructure further limit access to VCT services within schools. These findings provide a roadmap for designing youth-centered VCT programs that tackle stigma, enhance confidentiality, and improve rural access, significantly advancing HIV prevention in Tanzania.

## Supplementary material

10.2196/66739 Checklist 1 STROBE checklist.

## Abbreviations

UNAIDS: The Joint United Nations for Acquired Immune Deficiency Syndrome
VCT: voluntary counseling and testing
