# Supplementary material for: Knowledge, Perception, and Attitude Toward Voluntary Counseling and Testing for HIV Among Secondary School Students in Iringa Rural District: Descriptive Cross-Sectional Study
Source: JMIR Public Health Surveill. 2025 Sep 26;11:e66739. doi: 10.2196/66739 (PMC12519304; doi:10.2196/66739)
Supplement: Checklist 1 [file publichealth-v11-e66739-s001.pdf]

STROBE Statement—Checklist of items that should be included in reports of ***cross-sectional studies***

	Item No	Recommendation	Page No
**Title and abstract**	1	Knowledge, Perception, and Attitude Towards Voluntary Counselling and Testing For HIV/AIDS Among Secondary School Students in Iringa Rural District: A Descriptive Cross-Sectional Study.	1
		**Abstract**<br>**Background;** Voluntary Counselling and Testing (VCT) for HIV/AIDS is characterized by several key components, including pre-test and post-test counselling, as well as the formulation of individualized risk reduction plans. Adolescents, including secondary school students, represent a population particularly vulnerable to HIV infection due to various biological, psychological, and social factors. In this context, the present study aimed to assess the knowledge, perceptions, and attitudes toward VCT for HIV/AIDS among secondary school students in Iringa Rural District, Tanzania.<br>**Methodology:** A descriptive cross-sectional study was conducted in Iringa Rural District, targeting secondary school students. A random sampling technique was employed to select participating schools. Data were collected through self-administered questionnaires, which were completed solely by students who voluntarily consented to participate in the study. Data management and analysis were carried out using Epi Info version 7 (Epi7) software.<br>**Results;** The study involved 127 secondary school students aged 15–25 years from three schools in Iringa Rural District, with 69 (54.3%) female and 58(45.7%) male participants. All students were aware of Voluntary Counselling and Testing (VCT) services. The primary source of VCT information was school-based education 92(33.3%), followed by radio/TV 65(23.6%), and friends/family 46(16.7%) and Magazines 35 (12.7%). Some	1

		students also cited health centers, hospitals, and religious seminars 38 (13.8%) as sources. Knowledge of VCT increased with education level, with Form IV students showing the highest awareness 67 (55.4%). Most students understood VCT's main purpose: 88(50.6%) linked it to knowing one’s HIV status, 58(33.3%) to HIV prevention, and 28(16.1%) to preparing for test results. No misconceptions about VCT’s purpose were reported. Attitudes toward HIV testing showed that stigma concerns increased with age, particularly among those aged 17–18, where 26(60.5%) were unwilling to be identified as HIV-positive. Nonetheless, 65(51.2%) of students supported knowing their HIV status, and 86(54.4%) said they would inform others and change their behaviour after testing. Key barriers to VCT uptake included fear of stigma 71 (50.6%), lack of confidentiality, insufficient trained personnel, and poor infrastructure. These findings highlight both progress and persistent challenges in VCT awareness and acceptance among rural Tanzanian adolescents.<br>**Conclusion:** The study found that secondary school students in Iringa Rural District had generally high awareness and positive attitudes towards Voluntary Counselling and Testing (VCT) for HIV, mainly informed through schools and media. Awareness increased with education level, but willingness to disclose test results remained low due to stigma and confidentiality concerns. While many students were ready to take responsible actions after testing, barriers such as fear, misinformation, and limited access to services persisted. The study emphasizes the need for improved school-based VCT programs, better-trained staff, community education, and strategies to address stigma and infrastructural gaps.	
**Introduction**			
Background/rationale	2	Since the year 2000, approximately 38.1 million individuals	3-5

		have contracted HIV, and about 25.3 million have lost their lives due to illnesses related to AIDS. Despite better access to antiretroviral therapy and healthcare in many parts of the world, the AIDS epidemic still claimed 1.2 million lives in 2014 alone, and globally, youth aged 15–24 account for nearly one-third of new HIV infections. Despite this, uptake of VCT services among youth remains low. The majority of these cases occurred in sub-Saharan Africa, which is home to 66.6% of all people living with HIV and most HIV transmissions happen through heterosexual sex, from mother to child during childbirth or breastfeeding, and through unsafe blood transfusions. Sub-Saharan Africa's burden is particularly evident in countries like Tanzania, where rural regions face distinct challenges. HIV/AIDS remains a major public health concern in Tanzania, with approximately 1.6 million people living with HIV as of 2023Therefore, understanding the knowledge, perception, and attitude towards VCT among secondary school students becomes crucial in designing effective interventions and strategies.	
Objectives	3	**Broad objective:** To assess the knowledge, perception and attitude towards voluntary counselling and testing for HIV/AIDS among secondary school students in Iringa rural district.<br>**State specific objectives:**<br>1.To determine the knowledge of secondary school Students towards VCT for HIV/AIDS in Iringa rural district.<br>2.To determine the attitudes of secondary school students towards VCT services for HIV/AIDS in Iringa rural district.<br>3.To determine the perceptions of secondary school students on VCT services for HIV/AIDS in Iringa rural district.<br>4.To determine the barriers towards use of VCT services for HIV/AIDS among secondary school students in Iringa rural district.	6
**Methods**			
Study design	4	This was A descriptive cross-sectional study	6
Setting	5	This study was done at three secondary schools (Lipuli, Kalenga and Kidamali) in Iringa rural district. Iringa Rural District is one of the four districts of the Iringa Region of Tanzania, East Africa. It is bordered to the north by the Dodoma Region, to the east by Kilolo District and encircles Iringa Urban District, to the south by the Mufindi District, to the southwest by the Mbeya Region and to the northwest by the Singida Region. Iringa district has a cold climate which favours agricultural activities and livestock keeping, thus majority of the people are peasant farmers. Original	6

		occupants are Hehe tribe but due to migration of people, the district has been occupied with many people of different ethnicity.	
Participants	6	The target population was secondary school students in three secondary schools which were randomly selected and comprised both males and females aged between 15-25 years. This study excluded students who were below 15 years old and above 25 years old, as well as those who declined to participate in the study.	6
Variables	7	Age criteria was made by (15-25) years; Gender (males and females).<br>Knowledge was defined by encompasses understanding the modes of transmission, prevention strategies, and the benefits of early diagnosis<br>Perception and attitude towards VCT are influenced by various factors, including cultural, religious, and social norms. Stigma and discrimination associated with HIV/AIDS can hinder individuals from seeking testing services due to fear of being judged or ostracized.	7
Data sources/ measurement	8	The data were collected from secondary school students using self-administered questionnaires. The purpose of the study was fully disclosed to the participants, allowing them to make an informed decision about their participation. Knowledge was assessed by if they are aware of VCT with Yes or No responses. Also, they were asked the sources of information about VCT (Friends/Family, taught in school, Radios/TV, Magazine, or others). Attitude was assessed by their willingness for HIV testing. Perception was assessed by their action after testing results for their HIV status.	7
Bias	9	Their questions were addressed. Participants were informed that their involvement in the study was voluntary. To ensure anonymity, participants were instructed not to provide their names on the questionnaire forms. Confidentiality was also assured, with individual consent and confidentiality guarantees given.	7
Study size	10	Sample size was calculated as follows:<br>$N = Z^2 P Q/E^2$, N= minimum sample size, Z= standard normal deviation, usually set at 1.96 which Corresponds to 95% confidence interval, P=the prevalence of HIV/AIDS in Iringa region (9.1%), Q= (1 – P) E, standard error of estimate=0.05; Therefore, the sample size required was 127.	7
Quantitative variables	11	Quantitative variables were handled by Coding, entry, cleaning and analysis was done using Epi Info™ 7.2 software.	8
Statistical methods	12	Descriptive statistics included Age, Gender, and level of education.<br>Knowledge was determined by Level of education, sources	8

		of information about VCT services and Importance of VCT services.<br>Attitude and perception were determined by readiness for to be known for your HIV status, Willing of secondary school students to disclose the HIV test results and Acceptability of secondary school students towards HIV test.<br>Perception was determined by Action to be taken by secondary school students after having tested for HIV, and Student reaction if tested positive for HIV.	
**Results**			
Participants	13	Secondary schools involved in our study were randomly selected from the number of selected schools in Iringa rural district.<br>**Inclusion criteria:** Secondary school students, aged between 15-25years and willing to be interviewed and participate in the study.<br>**Exclusion criteria:** This study excluded students who were below 15 years old and above 25 years old, as well as those who declined to participate in the study.	8
Descriptive data	14	A total of 127 students from three secondary schools aged 15-25 years were enrolled in the study. Out of the 127 students, 69 (54.3%) and 58 (45.7%) students were females and males respectively. All the 127students were aware about VCT services. The source of information on VCT for HIV/AIDS among students was determined. The primary sources of information, that involved describing whether have heard of VCT or not it was revealed that the source of information were schools, accounting for 92(33.3%) of the participants. Radios/TV were the second common source, with 65 (23.6%) of participants obtaining information from this medium. Friends/family were another significant source, with 46(16.7%) of participants relying on them. Magazines were the least utilized source, with only 35(12.7%) of participants obtaining information from them. On the other hand, approximately 38(13.8%) of participants reported obtaining information from Health centres, Hospitals, mosques, and church seminars	8-10
Outcome data	15	A total of 127 students from three secondary schools aged 15-25 years were enrolled in the study. Out of the 127 students, 69 (54.3%) and 58 (45.7%) students were females and males respectively. All the 127students were aware about VCT services. The primary sources of information, that involved describing whether have heard of VCT or not it was revealed that the source of information were schools, accounting for 92(33.3%) of the participants. Radios/TV were the second common source, with 65 (23.6%) of participants obtaining information from this medium. Friends/family were another significant source, with	11-19

		46(16.7%) of participants relying on them. As puberty stage approaches, attitude towards readiness to have HIV test also decreases as this was seen among 12(44.4%) and 26(60.5%) students with the age range (17-18 years) respectively who were strongly not ready to know their HIV status among their secondary school students. Willingness of secondary students towards disclosure of HIV test was determined. 43(33.7%) students strongly disagreed to be known for their HIV status after HIV test while 27(21.3%) strongly disagreed to be known their HIV status. Perception of secondary students towards VCT services for HIV/AIDS was determined. Most of secondary students 86(54.4%) and 50 (31.7%) agreed that actions to be taken were to tell their parents or close friends and to change their behaviours respectively after HIV test. Reaction of secondary students after having tested positive for HIV was determined. Large proportion of students 122(64.2%) were ready to follow doctor's advice while only 1(0.5%) student would commit suicide after testing positive for HIV. Barriers among secondary school students towards utilization of VCT services was determined. The barriers identified were social discrimination and stigmatization as more as students expressed concerns that those who tested positive for HIV could face discrimination from their community members, compounded by lack of confidentiality among health care providers, low number of health care providers and low count of infrastructures for VCT services<br>**Summary measures**: It is important to educate secondary school students that they are not too young to be at risk for HIV/AIDS. As they are in a sexually active age group, they should be made aware of the importance of HIV testing. Parents should also be educated about VCT services and encouraged to have open discussions about it with their children. Media outlets should be encouraged to increase educational sessions on the significance of VCT services for HIV/AIDS and related illnesses. Secondary school students should be motivated to undergo HIV testing. Lectures on VCT services should continue to be included in the secondary school syllabus. The Tanzania government, through the Ministry of Health, should increase the number of personnel to facilitate the use and operation of VCT services for this young population.	
Main results	16	**Characteristics of the study participants:**<br>A total of 127 students from three secondary schools aged 15-25 years were enrolled in the study. Out of the 127 students, 69 (54.3%) and 58 (45.7%) students were females and males respectively. All the 127students were aware about VCT services. The source of information on VCT for	11

HIV/AIDS among students was determined. The primary sources of information, that involved describing whether have heard of VCT or not it was revealed that the source of information were schools, accounting for 92(33.3%) of the participants. Radios/TV were the second common source, with 65 (23.6%) of participants obtaining information from this medium. Friends/family were another significant source, with 46(16.7%) of participants relying on them. Magazines were the least utilized source, with only 35(12.7%) of participants obtaining information from them.

Knowledge towards the importance of VCT services

The proportion of students with knowledge on the importance of VCT services increased with increased Level of Education. Out of 127 students, where the proportion of form II, III, and IV were 8(6.61%), 46(38.0%) and 67(55.4%) respectively. The majority of students who were aware of Voluntary Counselling and Testing (VCT) for HIV/AIDS understood that the primary purpose of VCT is to determine one's HIV status which involved 88 (50.6%) of students while 58 (33.3%) students knew that VCT services help people to prevent HIV prevention**,** and 28(16.1%) students responded that VCT services prepares an individual to accept HIV test results.

Attitudes of participants towards VCT services of secondary school students

As puberty stage approaches, attitude towards readiness to have HIV test also decreases as this was seen among 12(44.4%) and 26(60.5%) students with the age range (17-18 years) respectively who were strongly not ready to know their HIV status among their secondary school students. Acceptability towards HIV test for secondary students was also determined which revealed 65 (51.2%) students reasoned that it’s compulsory to know their HIV status while only 6 (4.7%) students showed that, no necessity to know HIV status. Willingness of secondary students towards disclosure of HIV test was determined. 43(33.9%) students strongly disagreed to be known for their HIV status after HIV test while 27(21.3%) strongly disagreed to be known their HIV status.

Perception of participants towards VCT services of secondary school students

Perception of secondary students towards VCT services for HIV/AIDS was determined. Most of secondary students 86(54.4%) and 50 (31.7%) agreed that actions to be taken were to tell their parents or close friends and to change their behaviours respectively after HIV test. Reaction of secondary students after having tested positive for HIV was determined. Large proportion of students 122(64.2%) were

		ready to follow doctor’s advice while only 1(0.5%) student would commit suicide after testing positive for HIV.<br>**Barriers towards VCT services among secondary school students:**<br>Barriers among secondary school students towards utilization of VCT services was determined. Most students 71 (50.57%) responded that stigmatization from the society was the great obstacle to have HIV test while 26(18.6%) students said that they will die early, on the other hand 25(17.9%) students said that still lack of confidentially exist among health care works and making them harder to know their HIV statuses.<br>Reasons for lack of coverage for VCT services for secondary school students were determined. A larger proportion of students 46(56.8%) said it’s due to lack of personnel for VCT services while 17(21.0%) students said, it’s of because of poor infrastructure.<br>Knowledge of VCT and Information Sources	
Other analyses	17	None	
**Discussion**			
Key results	18	**Participant Characteristics and Implications for VCT Uptake**<br>This study enrolled 127 secondary school students aged 15–25 from three secondary schools, with a relatively balanced gender distribution, consistent with school-based demographic trends in rural Tanzania. The majority were aged 17–18 years, suggesting that mid-adolescence is the key period for interventions promoting Voluntary Counselling and Testing (VCT) services. This age group typically experiences increased autonomy, sexual debut, and curiosity—factors that elevate HIV risk and underline the importance of early preventive strategies. Similar demographic trends were found in a study conducted by Mkumbo and Ingham (2010) in Mwanza [20], which emphasized that students aged 16–18 were more receptive to sexual and reproductive health interventions due to greater maturity and exposure to Sexual Reproductive Health (SRH) topics in school curricula. This supports the current finding that students in higher academic levels (Form III and IV) showed greater awareness and more positive attitudes towards VCT, emphasizing the educational system’s critical role in shaping health behaviors.<br>**Knowledge of VCT and Information Sources**<br>All students reported awareness of VCT services, and schools were identified as the main source of this information, followed by radio/TV and friends/family. This	18-23

aligns with findings by Anastasia et al. in Uganda, where school-based interventions were the leading source of HIV-related knowledge among adolescents [21]. The prominence of mass media as a secondary source supports the notion that integrating media campaigns with school-based programs can reinforce HIV prevention messages. However, the low reliance on interpersonal sources like family or health centers suggests a missed opportunity for community-based health promotion. This is consistent with Ndeki et al. who reported that youth often perceive HIV as a "school topic" rather than a family concern, emphasizing the need to involve parents and local healthcare workers in sensitization campaigns to bridge this communication gap [22].

Awareness and Perception of the Importance of VCT

Awareness of the importance of VCT improved with educational level, with Form IV students demonstrating the highest levels of awareness. This echoes findings from Mboya et al. in Southern Highlands, Tanzania, where older students and those in upper forms exhibited significantly higher knowledge and appreciation of VCT's role in HIV prevention and care [25]. Students primarily cited knowing one's HIV status and preventing HIV as key reasons for seeking VCT, indicating a sound understanding of VCT's core objectives.

Nevertheless, the relatively low mention of emotional preparedness reflects a gap in psychological aspects of VCT counselling. Comprehensive VCT services should therefore include components that prepare youth emotionally, not just medically, for their HIV test results.

Attitudes Towards HIV Testing and Disclosure

The majority of students expressed positive attitudes towards HIV testing, with 51.2% strongly agreeing and 31.5% agreeing to be tested. However, only 38.6% were willing to disclose their HIV test results, while 55.2% disagreed or strongly disagreed. This dichotomy reflects a tension between individual health behaviour and social repercussions, likely fuelled by ongoing stigma. Comparable concerns were found in Wandera et al. in Kenya, where stigma was a major deterrent to disclosure despite high testing willingness [26].

The 17–18-year-old group exhibited the highest acceptance of being known for their HIV status, suggesting this group may serve as peer educators or ambassadors in youth-centered VCT campaigns. On the contrary, 15–16-year-olds showed higher levels of uncertainty and fear—highlighting the importance of age-specific sensitization.

Perception of VCT Outcomes and Behaviour Change

		In terms of anticipated post-test behaviour, the most common actions were informing parents or close friends and changing risky behaviour. These findings indicate a generally positive perception of VCT as a preventive and supportive service. However, the low number of students indicating condom use as a post-test action suggests persistent gaps in knowledge or cultural taboos around condom use. A study by Matasha et al. in rural Morogoro found that misconceptions and socio-cultural norms significantly deterred condom acceptance, even among informed youth [27]. The vast majority of students said they would follow doctors' orders if tested HIV-positive, which is encouraging.<br>**Barriers towards utilization of VCT Services by secondary school students**<br>In this study, Stigma emerged as the most reported barrier, consistent with nearly all recent adolescent HIV studies in sub-Saharan Africa. For instance, Mugisha et al. in Uganda reported that perceived stigma was the leading reason adolescents avoided VCT services, despite widespread awareness [30]. In the current study, students also cited fear of dying and concerns about confidentiality, indicating that both misinformation and systemic trust issues with healthcare workers remain significant challenges. These findings support the need for community-wide de-stigmatization campaigns and robust training for healthcare providers in confidentiality. Similar findings were found by Vulstan Shedura et al [23], Emmanuel Anongeba Anaba et al [31], and Lema Abate Adulo et al [32]. Lack of confidentiality among healthcare provider was also hindering factor for secondary school students towards VCT services utilization, while other have the notion that they are too young, therefore they cannot get HIV, which in fact contribute to the continuous prevailing of high spread of HIV infection among young population in the Iringa region. Our study also identified a lack of trained personnel and poor infrastructure as major constraints to VCT accessibility. These are structural barriers that mirror findings from UNAIDS (2023) which stressed that youth-friendly HIV services are severely under-resourced in rural areas. Efforts to decentralize and strengthen VCT services in school environments are therefore paramount [33].	
Limitations	19	The study was conducted in three secondary schools in a single district in a region, which may limit generalisability. Future research is needed to cover large geographical population of multiple regions along the country, compounded with large sample size.	23
Interpretation	20	1.School-Centered Interventions Are Effective: The high	23-

		levels of awareness and acceptance of VCT among Form III and IV students affirm the value of integrating VCT education into school curricula. Education ministries should consider expanding such programs to lower forms to reach younger adolescents early.<br>2.Addressing Stigma and Confidentiality: The reluctance to disclose HIV status and the perceived breach of confidentiality point to urgent needs for stigma-reduction campaigns and the implementation of trusted, youth-friendly VCT centers with strict confidentiality policies.<br>3.Targeted Behavioural Change Communication: The limited mention of condoms as a preventive method and misconceptions about HIV's fatality indicate a need for targeted behavioural interventions that challenge myths and promote accurate knowledge, especially around ART, prevention, and healthy living.<br>4.Engaging Families and Communities: Since interpersonal sources like families and health workers play a minor role in VCT education, strategies should involve parents, religious leaders, and community health workers to normalize discussions about HIV and reduce shame.<br>5.Mental Health Integration: The instance of suicidal ideation, underscores the need to include psychosocial support services within VCT frameworks. Adolescents require reassurance and coping mechanisms to handle positive diagnoses constructively.<br>6.Resource Allocation and Infrastructure: The findings highlight that for VCT programs to scale up in rural districts, investments are needed in human resources, mobile clinics, and infrastructure to bridge current service delivery gaps.	24
Generalisability	21	The study was conducted in three secondary schools in a single district in a region, which may limit generalisability.	23
**Other information**			
Funding	22	The authors declared that this study has received no financial support.	26
